# Social Context, Stress, Neuropsychiatric Disorders, and the Vasopressin 1b Receptor

**DOI:** 10.3389/fnins.2017.00567

**Published:** 2017-10-16

**Authors:** Heather K. Caldwell, Elizabeth A. Aulino, Karla M. Rodriguez, Shannah K. Witchey, Alexandra M. Yaw

**Affiliations:** ^1^Laboratory of Neuroendocrinology and Behavior, Department of Biological Sciences Kent State University, Kent, OH, United States; ^2^School of Biomedical Sciences, Kent State University, Kent, OH, United States

**Keywords:** social recognition memory, aggression, neuropsychiatric disorders, hormonal stress response, animal models

## Abstract

The arginine vasopressin 1b receptor (Avpr1b) is involved in the modulation of a variety of behaviors and is an important part of the mammalian hormonal stress axis. The Avpr1b is prominent in hippocampal CA2 pyramidal cells and in the anterior pituitary corticotrophs. Decades of research on this receptor has demonstrated its importance to the modulation of social recognition memory, social forms of aggression, and modulation of the hypothalamic-pituitary-adrenal axis, particularly under conditions of acute stress. Further, work in humans suggests that the Avpr1b may play a role in human neuropsychiatric disorders and its modulation may have therapeutic potential. This paper reviews what is known about the role of the Avpr1b in the context of social behaviors, the stress axis, and human neuropsychiatric disorders. Further, possible mechanisms for how Avpr1b activation within the hippocampus vs. Avpr1b activation within anterior pituitary may interact with one another to affect behavioral output are proposed.

## Introduction

It is well-established that the neuropeptide arginine vasopressin (Avp) is important to the neural modulation of mammalian behavior. However, the nuances of how Avp modulates behavior within specific brain regions via its two centrally expressed receptors, the Avp 1a (Avpr1a) and the Avp 1b receptor (Avpr1b), continues to be a robust and exciting area of research. While the Avpr1a has been heavily studied for several decades, the Avpr1b, which was discovered later, appears to be much more discretely localized and has a wholly different role in the modulation of behavior than the Avpr1a.

Since the initial papers describing the cloning of the Avpr1b (Lolait et al., 1995; Saito et al., 1995), its importance to the neural regulation of social behavior and the modulation of the hormonal stress response has come to light. Prominently expressed in the CA2 region of the hippocampus as well as the anterior pituitary gland (Young et al., 2006), Avp signaling through the Avpr1b can affect numerous behaviors, including social memory and aggression. Within the CA2 region of the hippocampus, the Avpr1b is hypothesized to be important for the processing of chemosensory information associated with social context, which in turn affects behavioral output (Stevenson and Caldwell, 2012; Pagani et al., 2015). Avpr1b expression in the anterior pituitary corticotrophs helps synergize the Avp signal with corticotropin releasing hormone (CRH) to facilitate the release of adrenocorticotropic hormone (ACTH). In fact, depending on the type of stressor, Avp signaling through the Avpr1b can have more of an impact on ACTH release than CRH (Ma et al., 1997, 1999). While the aforementioned roles of the Avpr1b may seem disparate, there are important possible points of intersection. For instance, the stress response under both acute and chronic conditions can result in changes in anxiety or mood (Roper et al., 2011), ultimately shaping how an organism might interpret its social world, in turn affecting social behavior. So, while central signaling of Avp via the Avpr1b is often considered distinct from its pituitary action, it is important to consider how they may interact. Given the complexity of the central Avp system, as well as the many behaviors Avp is known to affect, this review will focus on the role of Avp signaling via the Avpr1b in the modulation of behaviors such as social memory and aggression as well as the importance of this system to the functioning of the hypothalamic-pituitary-adrenal (HPA) axis. Lastly, the role of the Avpr1b in humans and the potential implications of this work in the context of neuropsychiatric disorders will be explored.

## The Avpr1b is important for social recognition memory

One critical component of social context is the ability of an animal to remember conspecifics, termed social recognition memory. Social memory also contributes to social cognition, which essentially requires an animal to remember a conspecific that they have interacted with previously. The ability to remember is key to helping an animal decide whether they should engage or avoid an interaction. The broader social context is also important to social memory. For example, the presence of other conspecifics or predators may impact an animal's choice to engage in certain behaviors, or even interfere with memory formation. As mentioned previously, there is compelling evidence that the Avpr1b is important for social recognition memory, specifically, its acquisition (Smith et al., 2016). This conclusion is based on studies utilizing Avpr1b knockout (^−/−^) mice, as well as excitotoxic lesions and optogenetic activation of the CA2 region of the hippocampus.

Across a variety of tests, Avpr1b^−/−^ mice display deficits in social recognition memory, despite having normal olfaction (Wersinger et al., 2002, 2004). In an 11-trial habituation/dishabituation task Avpr1b^−/−^ males show normal habituation and dishabituation to stimulus females—mice should decrease interaction times (as measured by proximity to the stimulus animal) across trials 1–10 when exposed to the same mouse repeatedly, i.e., habituation, and then increase their interaction time when exposed to a new mouse on trial 11, i.e., dishabituation. However, the durations of their investigation times are significantly lower in several of the trials compared to controls. These results suggest that Avpr1b^−/−^ mice are able to habituate to a familiar female and are able to recognize a novel female. However, the decreases in time spent investigating the stimulus mouse compared to controls could be indicative of decreased social motivation.

Consistent with this hypothesis, Avpr1b^−/−^ mice commonly demonstrate deficits in interacting with social stimuli (Wersinger et al., 2004; Yang et al., 2007; DeVito et al., 2009). Generally speaking, Avpr1b knockouts prefer a novel mouse over an inanimate object (Yang et al., 2007); although, in this particular study one cohort of null mutant and heterozygous Avpr1b mice failed to spend more time in the chamber housing the novel mouse relative to the chamber with the novel object. Additionally, Avpr1b knockouts spend less time with a familiar mouse vs. an empty compartment compared to controls (DeVito et al., 2009). Avpr1b^−/−^ mice also differ from wildtype controls in an olfactory social investigation task in which mice are exposed to male, female, and clean bedding in three trials such that preference for (1) male or female, (2) female or clean, and (3) male or clean bedding are assessed (Wersinger et al., 2004). While control animals exhibit the expected preference for female over male bedding and soiled (male or female) bedding over clean, Avpr1b^−/−^ mice display no preference for any type of bedding, which too suggests decreases in social motivation.

**Figure 1:**
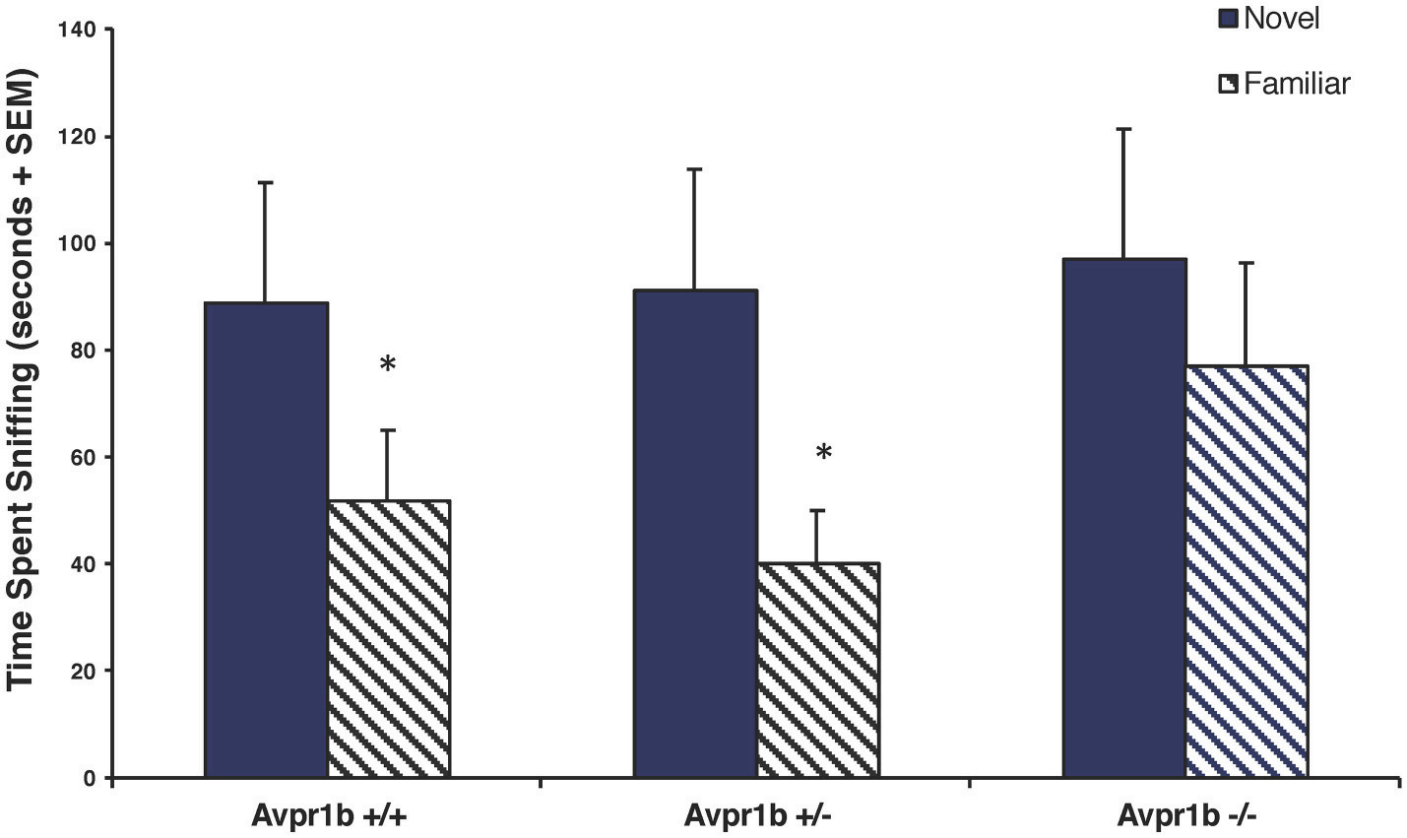
Social recognition is impaired in Avpr1b^−/−^ males as compared with their wild-type (Avpr1b^+/+^) and heterozygous (Avpr1b^+/−^) littermates. In a social recognition test with a 30-min interval between trials, Avpr1b^−/−^ males do not appear to recognize a familiar female, compared to controls. Data are expressed as mean + SEM. ^*^Significantly less than first exposure, *P* < 0.05. Modified and reprinted from Wersinger et al. (2002) with permission from Nature Publishing Group.

While Avpr1b knockout mice can habituate/dishabituate to social stimuli when there are short intertrial intervals, knockouts display memory deficits in more challenging tasks requiring temporal memory. When tested in the 2-trial social recognition test, which requires an animal to discriminate between a novel and a familiar animal with a 30-min intertrial interval, Avpr1b^−/−^ males have impaired social recognition, as they are not able to discriminate between a novel and a familiar female (Wersinger et al., 2002; DeVito et al., 2009; Figure 1). Interestingly, Avpr1b^−/−^ males do not seem to have any deficits in spatial memory (Wersinger et al., 2002; DeVito et al., 2009), but do exhibit impairments in two different tasks assessing temporal memory. In a “when” task that asks mice to discriminate between familiar objects presented at different time points as well as in an object-trace-odor task where mice are asked to learn associations with odors, Avpr1b^−/−^ mice fail to recall or integrate the associations after a time delay (DeVito et al., 2009). Thus, it appears that genetic disruption of the Avpr1b can compromise an animal's ability to retain the memory of a conspecific beyond a short period of time.

While much of the work to date has focused on males, since females' behaviors are often not as robust on some of the aforementioned tasks, there is evidence that female Avpr1b^−/−^ mice may also have deficits in social recognition memory. Specifically, female Avpr1b^−/−^ mice have an abnormal Bruce effect (Wersinger et al., 2008). The Bruce effect is a pheromonally-mediated response in which a female will abort her pregnancy, i.e., pregnancy block, following the presentation of a novel male or novel male odor (Bruce and Parrott, 1960). Interestingly, unlike controls, Avpr1b^−/−^ females fail to terminate their pregnancies in the presence of an unfamiliar male (Wersinger et al., 2008). Thus, Avpr1b^−/−^ females are not able to identify the unfamiliar male as being “new,” which is consistent with the hypothesis that the Avpr1b is important for processing olfactory cues, including accessory olfactory cues, which help the animal determine its social context and ultimately its behavioral response.

While studies that have utilized knockout mice have provided critical insight into the role of this receptor, one of the shortcomings of traditional knockout mice is that the gene is absent throughout the body from the point of fertilization, which in turn could result in some sort of developmental compensation. Fortunately, lesion studies and optogenetic work have confirmed much of what has been observed in Avpr1b^−/−^ mice. These studies have further implicated the CA2 region of the hippocampus. More importantly, Avpr1b expression within the CA2 region was confirmed to play a role in the neural modulation of social behavior. When the CA2 region of the hippocampus is excitotoxically lesioned in males, social recognition memory is impaired in both the 2-trial social discrimination test and 11-trial habituation/dishabituation social recognition test (Stevenson and Caldwell, 2014). Further, targeted Cre-driven viral inactivation of CA2 pyramidal neurons results in a loss of social memory and a decrease in preference for social novelty. However, this inactivation does not impact sociability, as the mice still prefer a familiar littermate over an empty chamber (Hitti and Siegelbaum, 2014). During the acquisition phase of a social memory task (but not its retrieval phase), optogenetic stimulation of the Avp projection that originates in the paraventricular nucleus and extends to the CA2 region of the hippocampus, increases social recognition memory indefinitely. This effect on social memory is blocked when the Avpr1b antagonist SSR149415, also referred to as Nelivaptan, is injected into the CA2 region of the hippocampus (Smith et al., 2016). Taken together, these data provide compelling evidence that the deficits in social memory observed in Avpr1b^−/−^ mice are likely due to Avp action through the Avpr1b within the CA2 region of the hippocampus.

## The Avpr1b is important for normal aggressive behavior

Competitive behaviors, such as aggression are important for social bonds between conspecifics. That said there are significant sex differences in the hormonal and neural regulation of aggression. Intermale aggression, for instance, is androgen-dependent in rodents. Whereas, in female rodents, aggressive behaviors are primarily observed in post-parturient females, being rarely observed in virgin females. However, whether male or female, aggressive behavior is characterized by both offensive and defensive elements and is commonly evaluated using a resident-intruder test for territorial aggression.

The Avp system, particularly its signaling through the Avpr1a, is consistently implicated in the modulation of aggressive behaviors (Ferris et al., 1997, 2006). However, even with its more limited distribution, the Avpr1b also appears to be important for normal displays of aggression in rodents. In hamsters for instance, oral administration of the Avpr1b antagonist SSR149415 at both 10 and 30 mg/kg doses significantly reduces the duration of resident male hamsters' frequency and duration of offensive sideways behaviors, olfactory investigation, chase behaviors, and flank marking compared to the vehicle and the 1 mg/kg dose groups (Blanchard et al., 2005). Similarly, in mice, oral administration of SSR149415 reduces the duration of offensive aggression in a resident-intruder test and decreases the number of defensive bites in those forced to encounter a threatening predator (Griebel et al., 2003). Conversely, in lactating Wistar rats neither intracerebroventricular nor site specific (MPOA and BNST) infusion of SS149415 10-min prior to a maternal defense test have an effect on aggressive behaviors (Bayerl et al., 2014, 2016). However, it is possible that this lack of effect is due to the use of virgin female Wistar rats as stimulus animals, though it is important to note that lactating Long Evans rats have been shown to attack female intruders more than male intruders (Haney et al., 1989). Thus, it is also plausible that this represents a species-specific effect of this antagonist. It is also important to acknowledge that SSR149415 has previously been shown to have a high affinity for the human oxytocin receptor (OXTR) and could have affinity for the rodent Oxtr as well (Griffante et al., 2005). That said, data from Avpr1b^−/−^ mice support the assertion that the Avpr1b is important for aggressive behavior within both sexes.

**Figure 2:**
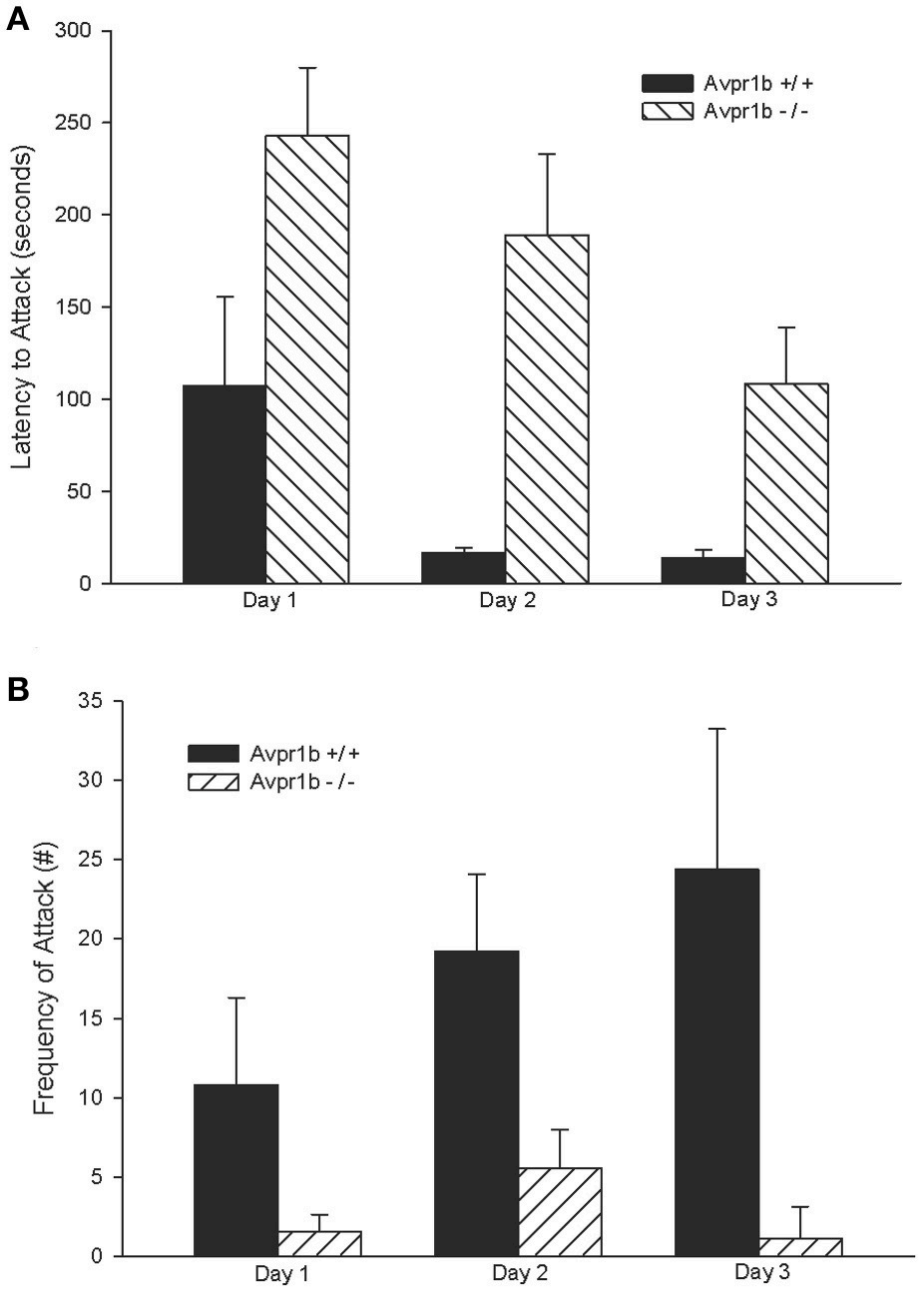
Intermale aggression is impaired in Avpr1b^−/−^ males on a more “wild” background, i.e., a 50/50 mixture of *Mus musculus* and *Mus musculus castaneus*, as compared with their wild-type (Avpr1b^+/+^) littermates. In a resident-intruder test Avpr1b^−/−^ males have longer attack latencies compared to Avpr1b^+/+^ mice **(A)**. Avpr1b^−/−^ mice also display fewer attacks compared to Avpr1b^+/+^ mice **(B)**. Data are expressed as mean + SEM. For **(A,B)** there is a main effect of day and genotype, but no interaction, *P* < 0.05. Modified and reprinted from Caldwell and Young (2009) with permission from Elsevier.

Compared to wildtype controls, Avpr1b^−/−^ males have lower attack frequencies, longer attack latencies, and Avpr1b^−/−^ males that do display aggressive behaviors display fewer agonistic behaviors (Wersinger et al., 2002, 2004). These observed reductions in aggressive behavior also extend to other mouse strains, with reduced aggressive behaviors persisting in Avpr1b^−/−^ males that are crossed with the more aggressive *Mus musculus castaneus* (Caldwell and Young, 2009; Figure 2). Importantly, these deficits in aggressive behavior are specific to social situations as Avpr1b^−/−^ males display normal predatory aggression (Wersinger et al., 2007). When Avpr1b^−/−^ males are used as intruders, to see if they will defend themselves against an attack, they display defensive postures in the absence of defensive attacks, but show fewer retaliatory attacks (Wersinger et al., 2007). This observation appears to hold true for females as well, with only 20% of lactating Avpr1b^−/−^ females displaying aggressive behaviors directed toward an intruder compared to 90% of lactating Avpr1b +/+ females. Further, of those lactating Avpr1b^−/−^ females that do attack, the latency to attack and number of attacks remain significantly lower compared to controls (Wersinger et al., 2007).

Of course, aggression is complex with different neural networks activated depending on the type of aggression. For example, defensive contexts activate the posteroventral medial amygdala and dorsomedial ventromedial hypothalamus, whereas in offensive contexts the posterodorsal medial amygdala appears to play more of a role (Swanson, 2000). For intermale and maternal aggressive behavior, many of the nodes within the social behavioral neural network (SBNN) have been identified as being important for their regulation (for review see, Nelson and Trainor, 2007). To determine which brain areas are important for the neural regulation of aggression in Avpr1b^−/−^ males and females, a couple of immediate early gene (IEG) studies have been performed. Two different IEGs, cFos and early growth response factor 1 (EGR1), have been studied in both Avpr1b^−/−^ male and Avpr1b^−/−^ lactating females following a single exposure to an intruder male. While no genotypic differences in cFos immunoreactivity were observed in either Avpr1b^−/−^ males or Avpr1b^−/−^ lactating females, a genotypic difference in EGR1 immunoreactivity was observed within the ventral bed nucleus of the stria terminalis (BNSTV) and the anterior hypothalamus (AHA), with male Avpr1b^−/−^ mice having reduced EGR1 immunoreactivity in both brain regions relative to controls (Wersinger et al., 2002; Witchey et al., 2016). As both the BNSTV and AHA are implicated in the neural circuitry of aggression, we hypothesize that they may be part of the downstream circuit influenced by Avpr1b expression in the CA2 region of the hippocampus.

Like social memory, CA2 Avpr1b is also known to directly affect aggressive behavior. When the Avpr1b is overexpressed via microinjection of a lentiviral vector into the dorsal CA2 region of Avpr1b^−/−^ males, their deficits in aggressive behavior are partially rescued (Pagani et al., 2015). Based on the work of Cui et al. (2013), it has been established that there is a Avp-ergic projection from the PVN to the CA2 region (this is what was driven in the aforementioned study by Smith et al., 2016). What happens downstream of the activation of Avpr1b within this region is still being determined. However, based on the connectivity of the CA2 region to other parts of the brain and what is known about the neural regulation of aggressive behavior a possible circuit can be hypothesized. The CA2 region has numerous efferent projections within the hippocampus (CA1, CA2, and CA3) as well as projections to the medial septum, the dorsal part of the LS, the triangular septal nucleus, the nuclei of the diagonal bands of broca, and the supramammillary nuclei (Chevaleyre and Siegelbaum, 2010). Of these, the projections to the septal regions are the most apparent link to the aggression circuit. Specifically, lesions or pharmacological inactivation of the LS leads to increased aggression (Slotnick et al., 1973; Potegal et al., 1981; McDonald et al., 2012) and conversely, electrical stimulation of the LS suppresses aggression (Potegal et al., 1981). Further, increases in cFos expression are observed within the LS following intermale, interfemale, and maternal aggression (Kollack-Walker and Newman, 1995; Delville et al., 2000; Davis and Marler, 2003; Hasen and Gammie, 2005). It is also important to mention that the LS lies upstream of the BNSTV and AHA (Ferris et al., 1990; Staiger and Wouterlood, 1990); thus providing a potential circuit that might explain the EGR-1 data in Avpr1b^−/−^ males (Witchey et al., 2016).

## Avpr1b receptors expressed in the pituitary corticotrophs play an important role in the stress response

**Figure 3:**
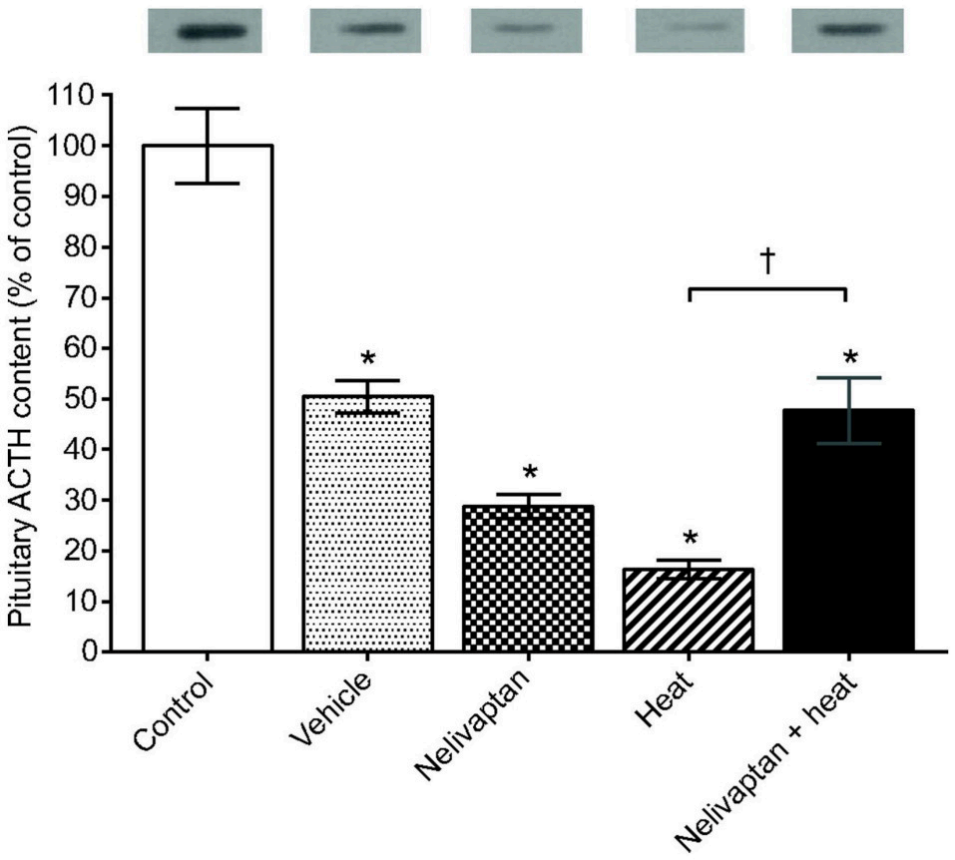
Pretreatment with SSR149145 (i.e., Nelivaptan) prior to heat exposure results in increased pituitary adrenocorticotropic hormone (ACTH), as measured by western blot. Data are expressed as mean ± SEM. ^*^A significant difference between the control and treated group of animals and ^*†*^A significant difference between treated groups, *P* < 0.05. The results of the western blot for ACTH are presented above each bar. Reprinted from Jasnic et al. (2013) with permission from Company of Biologists LTD.

Given its well-defined role as a critical regulator of the HPA-axis, the Avpr1b has been studied extensively in the context of the hormonal stress response. Most of these studies have utilized SSR149415 and in rats there is scientific consensus that administration of SSR149415 prior to a variety of stressors decreases plasma ACTH compared to controls (Serradeil-Le Gal et al., 2003; Chen et al., 2008; Zhou et al., 2011; Jasnic et al., 2013; Ramos et al., 2016). Evidence that pharmacological disruption of Avpr1b signaling affects CORT are less consistent and seem to depend primarily upon the type of stressor as well as the route of administration of SSR149415, as its biological activity can differ depending on how it is administered (Roper et al., 2011). For example, in male Wistar rats intracerebroventricular administration of SSR149415 before air jet stress results in decreases in heart rate, blood pressure, and CORT compared to untreated controls (Stojicic et al., 2008); the effects on ACTH in this study are not known as they were not measured. However, when SSR149415 is administered intravenously or orally to male Sprague–Dawley rats prior to noise or dehydration stress, respectively, they show decreases in their ACTH response but no change in CORT compared to controls (Chen et al., 2008). Interestingly, when SSR149415 is given intraperitoneally to male Wistar or Fischer rats before heat stress or cocaine withdrawal, respectively, there are significant decreases in both ACTH (Figure 3) and CORT compared to controls (Zhou et al., 2011; Jasnic et al., 2013). It is also important to note that none of the studies that have utilized SSR149415 have found that this antagonist can bring ACTH or CORT concentrations back to pre-stressor levels, indicating only a partial reversal. As noted earlier, cross-talk with the Oxtr also remains a possibility (Serradeil-Le Gal et al., 2003; Griffante et al., 2005; Oost et al., 2011), which further complicates the interpretation of these studies.

Beyond measures of HPA-axis function, studies that have utilized SSR149415 have also examined the effect of this antagonist on the expression of numerous stress phenotypes, primarily those with anxiety- or depressive-like features. In both rats and mice, treatment with SSR149415 results in fewer defensive attacks in a defense battery, less time spent immobile in a forced swim test, more foot shocks in a punished drinking task, and more open arm entries in an elevated plus maze (Griebel et al., 2002; Serradeil-Le Gal et al., 2003). Taken together, these data suggest that antagonism of the Avpr1b has an overall anxiolytic effect. This is consistent with studies in which the effects of Diazepam and SSR149415 have been directly compared, with SSR149415 having more potent anxiolytic and antidepressant effects during both punished drinking tasks and elevated plus maze tests, as compared to diazepam (Serradeil-Le Gal et al., 2003). While the aforementioned studies utilized only a single dose, data from a chronic dose study found similar effects. Specifically, Breuer et al. (2009) administered chronic doses of SSR149415 intraperitoneally to Sprague-Dawley rats who were hyperactive as a result of an olfactory bulbectomy. They found that after 14 days of treatment, SSR149415 was able to bring olfactory bulbectomy-related hyperactivity back to control levels, being as effective as imipramine. Further, this effect persisted for a week after the cessation of treatment, which suggests that SSR149415 may have long-lasting effects. Chronic treatment with SSR149415 has also been shown to reduce dysphoria, as measured by intracranial self-stimulation in rat nicotine-withdrawal models (Qi et al., 2015). Interestingly, Sprague-Dawley rats chronically administered SSR149415 into the dorsal hippocampus via minipump display decreases in anxiety-like behaviors in an elevated plus maze (Engin and Treit, 2008). This latter study hints at a possible point of intersection between the peripheral effects of the Avpr1b and those within the brain with regards to anxiety and mood.

Some of the lack of consensus regarding the effects of SSR149415 on the HPA-axis are likely due to real differences in how various stressors affect the HPA-axis. Variation in the timing of the data collection post stressor (Roper et al., 2011), as well as differences between rodent species and strains (Roper et al., 2010) are also important to consider. Another way to think about the mixed results would be to think about how SSR149415 affects the performance of the system as a whole. For example, an increase in peripheral Avp and an increase in Avpr1b protein expression within the pituitary has been observed when the Avpr1b is blocked by SSR149415, suggesting that Avpr1b expression in the pituitary is partially dependent on peripheral levels of Avp (Jasnic et al., 2013). Additionally, Ramos et al. (2016) found that the combination of SSR149415 and the CRH receptor antagonist SSR125543 was especially effective at lowering ACTH concentrations across three different types of stressors. Given that the Avpr1b and the CRH receptor type 1 are known to dimerize without impairing ligand binding and can modulate one another (Young et al., 2007; Murat et al., 2012), more research is needed to fully understand how SSR149415 is impacting signaling in this system.

Avpr1b^−/−^ mice too have provided insights into the role of this receptor in the mediation of the stress response. Avpr1b^−/−^ mice have normal resting ACTH levels but, as observed in the antagonist studies, following a variety of stressors, Avpr1b^−/−^ males have weaker ACTH responses (Tanoue et al., 2004; Lolait et al., 2007a,b; Stewart et al., 2008b; Roper et al., 2010). Complementing SSR149415 studies, there are also stressor-dependent differences in CORT responses in Avpr1b^−/−^ mice. For example, dehydration stress results in decreases in plasma CORT concentrations (Roberts et al., 2011). Male Avpr1b^−/−^ mice subjected to acute restraint and shaker stress have a blunted ACTH response (Stewart et al., 2008b; Roper et al., 2010) However, while males show a blunted ACTH response after forced swim test, CORT levels appear normal (Stewart et al., 2008b). Interestingly, female Avpr1b^−/−^ mice are more consistent in their ACTH and CORT response to stressors, showing reductions in both ACTH and CORT after lipopolysaccharide, ethanol, and selective serotonin reuptake inhibitor treatments (Lolait et al., 2007b; Stewart et al., 2008a).

With regards to stress phenotypes, the data are mixed in Avpr1b^−/−^ mice. No genotypic differences are reported for forced swim, chronic isolation, elevated plus, and open field tests (Wersinger et al., 2002; Caldwell et al., 2006; Itoh et al., 2006). Even in Avpr1b^−/−^ mice in which Avpr1b function in the CA2 region of the hippocampus is partially restored, no significant genotypic differences in anxiety-like behaviors are observed (Pagani et al., 2015). These results could be due to a compensatory mechanism, such as the upregulation of Oxtr in response to an absence of Avpr1b (Nakamura et al., 2008), but currently it is not clear why there is no obvious stress phenotype.

To better understand the role of Avpr1b in the mediation of stress behaviors, studies have focused on identifying the distribution of the Avpr1b and its local inhibition. In male Wistar rats, Avpr1b-associated immunoreactivity has been found in areas such as the amygdala, LS, nucleus accumbens, hippocampus, as well as others (Hernando et al., 2001). Of these areas, both the basolateral and medial amygdala have been implicated specifically in the mediation of anxiety by Avpr1b (Salome et al., 2006), with evidence for additional modulation via the Oxtr (Litvin et al., 2011), while the Avpr1b in the LS is suspected to be involved in depressive states (Stemmelin et al., 2005). In addition, infusions of SSR149415 into the dorsal hippocampus, amygdala, or LS of male Sprague-Dawley rats results in decreases in anxiety- and depressive-like behaviors, as measured by elevated plus or forced swim tests (Stemmelin et al., 2005; Salome et al., 2006; Engin and Treit, 2008; Zai et al., 2012). Even though none of these targeted infusion studies measured changes in ACTH or CORT levels, they do point to places in the brain where the peripheral and central effects of Avpr1b may interconnect. Further studies are required to fill the gaps in our understanding of the intersection of peripheral and central effects of Avpr1b.

## Data suggest that the Avpr1b may play a role in human neuropsychiatric disorders, emotional empathy, and have therapeutic potential

Much of the work implicating the Avpr1b in humans has emerged from genetic studies of single nucleotide polymorphisms (SNPs). Currently, there is evidence that SNPs within the Avpr1b sequence may impact social behaviors and aggression, as well as play a role in neuropsychiatric disorders, particularly those associated with dysregulation of the HPA-axis, such as mood and anxiety disorders (van West et al., 2004; Dempster et al., 2007; Keck et al., 2008; Zai et al., 2012). As reviewed above, animal models have linked the Avpr1b to the neural regulation of social recognition memory and aggression. Similarly, studies in humans have found Avpr1 SNPs to be involved in prosociality (Wu et al., 2015). It is well-established that in humans prosociality and empathy work hand in hand with each other; the former being a voluntary behavior exhibited to benefit others and the latter the ability to respond to others' emotions. For instance, carriers of the G allele of the Avpr1b SNP rs28373064 are more prosocial and empathetic, with the effects being mediated by emotional empathy rather than cognitive empathy (Wu et al., 2015). Furthermore, the c-allele of the AVPr1b SNP rs35369693 is associated with aggressive behavior in children aged 9–15 (Zai et al., 2012). Several other haplotypes have been reported that have yet to be genotyped, thus re-sequencing of the Avpr1b gene will be required to identify other possible variants and their association to childhood-onset aggression (Zai et al., 2012).

A common phenotype among patients with affective disorders is dysregulation of the HPA-axis (Dempster et al., 2007). In humans, variations in the Avpr1b gene have been found to be associated with mood disorders. van West et al. (2004) found that separate allele distributions along the 12-kb Avpr1b receptor gene are protective against recurrent major depression in a Swedish compared to Belgian adult population diagnosed with unipolar depression. Specifically, Avpr1b-s1, s2, s3, s4, and s5 SNP without a frequent G allele is protective in the Swedish population and Avpr1b-s5 SNP with a frequent G allele is protective in the Belgian population (van West et al., 2004). Consequently, the protective SNPs found in the Van West et al. study were used to investigate their involvement in childhood-onset mood disorders. In a study of Hungarian children diagnosed with a mood disorder prior to 15 years of age, genetic markers in the Avpr1b gene (rs28373064, rs35369693, and rs33985287) are directly associated with affective status in children. More importantly, this association is sex-specific, with these genetic markers being more common in females compared to males (Dempster et al., 2007). These findings are consistent with a study performed in twins that found that the heritability of depression is greater in females (42%) compared to males (29%) (Kendler et al., 2006).

Given that preclinical work in animal models suggests that antagonism of Avpr1b with SSR149415 can reduce anxiety-like and depressive-like behaviors (Griebel et al., 2002; Overstreet and Griebel, 2005), SSR149415 was approved for clinical trials. Unfortunately, to date, the data from the animal models does not appear to translate to humans. In a Phase II clinical trial in patients with major depressive disorder or generalized anxiety disorder, the effects of treatment with SSR149415 did not differ from the effects of the placebo (Roper et al., 2011; Griebel et al., 2012). Thus, further clinical studies are needed, likely with a different Avpr1b antagonist, to determine if manipulation of Avpr1b signaling may have some therapeutic benefit. Furthermore, the development of such a drug is likely to require genetic testing and biomarker identification to aid in identifying patients that are likely to be responsive to Avpr1b receptor antagonism.

## Integrative discussion

Clearly, the Avpr1b has an important, and conserved, function in the modulation of social behaviors as well as the hormonal stress response. Based on some very elegant work in preclinical models, as well as work in humans, it appears that at least one of the roles of the Avpr1b is to aid an animal in determining its social context. Plainly stated, social context is the physical and social setting in which an animal finds itself. Thus, the capacity of an animal to display an appropriate, context-specific, social behavior is often rooted in how that individual interprets their social environment. In the case of Avp signaling via the Avpr1b, the expression of the Avpr1b in the CA2 region of the hippocampus is hypothesized to be important for determining social salience, as its manipulation within this part of the brain impacts the acquisition of memories associated with social context as well as aggressive behaviors (Pagani et al., 2015; Caldwell and Albers, 2016; Smith et al., 2016). With regards to Avpr1b expression in the anterior pituitary, depending on the stressor, genetic disruption of the Avpr1b results in a blunted ACTH release compared to controls, but not always a reduced CORT response (Roper et al., 2011). Likewise, treatment with an Avpr1b antagonist has been found to reduce anxiety-like and depressive-like behaviors in rodents (Serradeil-Le Gal et al., 2005; Stevenson and Caldwell, 2012), and SNPs of the Avpr1b are associated with anxiety and depression in humans (van West et al., 2004; Dempster et al., 2007; Keck et al., 2008; Zai et al., 2012). As dysregulation of the HPA-axis can affect a variety of behaviors, including stress coping, this too shapes how an animal perceives its social environment and alters behavioral responses.

**Figure 4:**
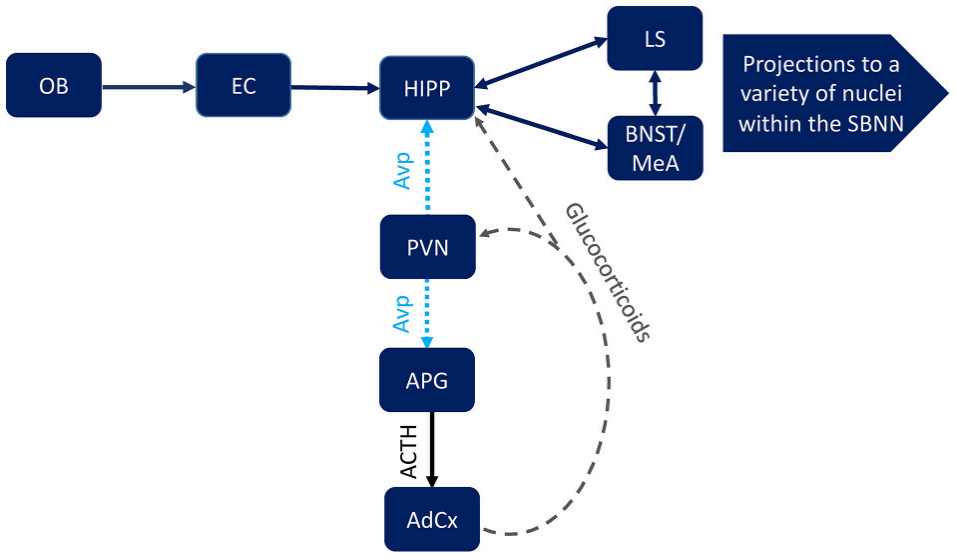
Possible points of intersection between hippocampal (HIPP) Avpr1b, which are localized to pyramidal cells in the CA2 region, and the mammalian stress axis, which includes Avpr1b in the anterior pituitary gland corticotrophs (APG). An animal's social context and subsequent behavioral responses to that context require a complex interaction between the social behavior neural network (SBNN), including the lateral septum (LS), bed nucleus of the stria terminalis (BNST), medial amygdala (MeA), as well as numerous other brain region. Input from the olfactory system, specifically the olfactory bulb (OB) and entorhinal cortex (EC), can be transmitted directly to the hippocampus (HIPP). The paraventricular nucleus (PVN) integrates external and internal information that can be conveyed to the Avpr1b via arginine vasopressin (Avp) projections to the HIPP and the APG (corticotropin releasing hormone release would also be stimulated). The result of the latter projection is activation of the stress axis, including adrenocorticotropic hormone (ACTH) release from the APG and subsequent glucocorticoid release from the adrenal cortex (AdCx). The glucocorticoids in turn act on numerous neural substrates in the brain, including the PVN and HIPP.

But how do these seemingly separate systems interact? We suggest that their interaction is dynamic and can be reinforcing. Specifically, it seems likely that the stress axis is affecting the interpretation of the social environment, but also that the social environment affects the stress axis. The CA2 region of the hippocampus represents a possible point of intersection of these two systems. It has already been established that the CA2 region is structurally and functionally distinct from other regions of the hippocampus (Lein et al., 2004, 2005). For instance, it is the only part of the hippocampus to receive input from the posterior hypothalamus (Borhegyi and Leranth, 1997; Vertes and McKenna, 2000; Bartesaghi et al., 2006) and the perforant pathway; which connects the entorhinal cortex to the hippocampal formation (Bartesaghi and Gessi, 2004). The entorhinal cortex receives input from the olfactory system, and its input into hippocampus is known to be important to the coding of olfactory-based memories (Petrulis et al., 2005; Sanchez-Andrade et al., 2005). This input to the hippocampus along with the Avp projection from the PVN may be involved in providing information about the social environment. Since the PVN is important for integrating numerous internal and external information and then serving as a control center that effects numerous autonomic functions, this seems plausible. But how would this occur? Perhaps via the glucocorticoid receptors that are expressed in the PVN, which are known to affect the expression of CRH, Avp (Sawchenko, 1987), as well as melanocortin receptors, i.e., MC3R (Roselli-Rehfuss et al., 1993). It is through these glucocorticoid receptors that the periphery could provide information about social context to Avp-ergic cells in the PVN, in turn altering Avp neurotransmission to the CA2 region. Likewise, input into the PVN directly affects the corticotrophs, which express the Avpr1b. Once the HPA axis is activated glucocorticoid receptors in the hippocampus (Reul and de Kloet, 1985; Aronsson et al., 1988; Arriza et al., 1988) may affect the input to, or from, the CA2 region (Figure 4). The interaction of these systems likely has wide-spread and context specific effects on neural targets, influencing a variety of behaviors, including anxiety-like, depression-like, and aggressive behaviors, which in turn has possible implications for numerous human neuropsychiatric disorders.

Based on what has been presented in this review it seems likely that the CA2 region represents a newly identified node in the SBNN, since this region appears to be a point of convergence for information about social context and perhaps social salience that then helps to influence behavioral output. The possibility that this brain area may represent a critical integrating site for where peripheral signals and the modulation of behavioral output occurs is quite exciting, but requires further study. That said, by improving our understanding of the connectivity of this system we may better understand species similarities and gain insights into, and improve therapeutics for, the numerous neuropsychiatric disorders that are characterized by abnormal sociability.

## Author contributions

All authors listed have made a substantial, direct and intellectual contribution to the work, and approved it for publication.

### Conflict of interest statement

The authors declare that the research was conducted in the absence of any commercial or financial relationships that could be construed as a potential conflict of interest.
